# CRITICS-II: a multicentre randomised phase II trial of neo-adjuvant chemotherapy followed by surgery versus neo-adjuvant chemotherapy and subsequent chemoradiotherapy followed by surgery versus neo-adjuvant chemoradiotherapy followed by surgery in resectable gastric cancer

**DOI:** 10.1186/s12885-018-4770-2

**Published:** 2018-09-10

**Authors:** Astrid E. Slagter, Edwin P. M. Jansen, Hanneke W. M. van Laarhoven, Johanna W. van Sandick, Nicole C. T. van Grieken, Karolina Sikorska, Annemieke Cats, Pietje Muller-Timmermans, Maarten C. C. M. Hulshof, Henk Boot, Maartje Los, Laurens V. Beerepoot, Frank P. J. Peters, Geke A. P. Hospers, Boudewijn van Etten, Henk H. Hartgrink, Mark I. van Berge Henegouwen, Grard A. P. Nieuwenhuijzen, Richard van Hillegersberg, Donald L. van der Peet, Heike I. Grabsch, Marcel Verheij

**Affiliations:** 1 Department of Radiation Oncology, Netherlands Cancer Institute - Antoni van Leeuwenhoek, Plesmanlaan 121, 1066 CX Amsterdam, The Netherlands; 2 Department of Medical Oncology, Academic Medical Center Amsterdam, Meibergdreef 9, 1106 AZ Amsterdam, The Netherlands; 3 Department of Surgery, Netherlands Cancer Institute - Antoni van Leeuwenhoek, Plesmanlaan 121, 1066 CX Amsterdam, The Netherlands; 4 Department of Pathology, VU University Medical Center, De Boelelaan 1117, 1081 HV Amsterdam, The Netherlands; 5 Statistical Department, Netherlands Cancer Institute - Antoni van Leeuwenhoek, Plesmanlaan 121, 1066 CX Amsterdam, The Netherlands; 6 Department of Gastroenterology, Netherlands Cancer Institute - Antoni van Leeuwenhoek, Plesmanlaan 121, 1066 CX Amsterdam, The Netherlands; 7 Department of Radiation Oncology, Academic Medical Center Amsterdam, Meibergdreef 9, 1106 AZ Amsterdam, The Netherlands; 8 Department of Medical Oncology, St. Antonius Hospital, Koekoekslaan 1, 3435 CM Nieuwegein, The Netherlands; 9 Department of Medical Oncology, St. Elisabeth Hospital, Hilvarenbeekse Weg 60, 5022 GC Tilburg, The Netherlands; 10 Department of Medical Oncology, Zuyderland Sittard-Geleen, Dr. H. van der Hoffplein 1, 6162 BG Sittard-Geleen, The Netherlands; 11 Department of Medical Oncology, University Medical Center Groningen, Hanzeplein 1, 9713 GZ Groningen, The Netherlands; 12 Department of Surgery, University Medical Center Groningen, Hanzeplein 1, 9713 GZ Groningen, The Netherlands; 13 Department of Surgery, Leiden University Medical Center, Albinusdreef 2, 2333 ZA Leiden, The Netherlands; 14 Department of Surgery, Academic Medical Center Amsterdam, Meibergdreef 9, 1106 AZ Amsterdam, The Netherlands; 15 Department of Surgery, Catharina Hospital Eindhoven, Michelangelolaan 2, 5623 EJ Eindhoven, The Netherlands; 16 Department of Surgery, University Medical Center Utrecht, Heidelberglaan 100, 3484 CX Utrecht, The Netherlands; 17 Department of Surgery, VU University Medical Center, De Boelelaan 1117, 1081 HV Amsterdam, The Netherlands; 18 Department of Pathology, GROW – School for Oncology and Developmental Biology, Maastricht University Medical Center, P Debyelaan 25, 6229 HX Maastricht, The Netherlands; 19 Department of Pathology & Tumour Biology, Leeds Institute of Cancer and Pathology, University of Leeds, Leeds, UK

**Keywords:** Gastric cancer, Resectable, Preoperative treatment, Chemotherapy, Chemoradiotherapy, Surgery

## Abstract

**Background:**

Although radical surgery remains the cornerstone of cure in resectable gastric cancer, survival remains poor. Current evidence-based (neo)adjuvant strategies have shown to improve outcome, including perioperative chemotherapy, postoperative chemoradiotherapy and postoperative chemotherapy. However, these regimens suffer from poor patient compliance, particularly in the postoperative phase of treatment. The CRITICS-II trial aims to optimize preoperative treatment by comparing three treatment regimens: (1) chemotherapy, (2) chemotherapy followed by chemoradiotherapy and (3) chemoradiotherapy.

**Methods:**

In this multicentre phase II non-comparative study, patients with clinical stage IB-IIIC (TNM 8th edition) resectable gastric adenocarcinoma are randomised between: (1) 4 cycles of docetaxel+oxaliplatin+capecitabine (DOC), (2) 2 cycles of DOC followed by chemoradiotherapy (45Gy in combination with weekly paclitaxel and carboplatin) or (3) chemoradiotherapy. Primary endpoint is event-free survival, 1 year after randomisation (events are local and/or regional recurrence or progression, distant recurrence, or death from any cause). Secondary endpoints include: toxicity, surgical outcomes, percentage radical (R0) resections, pathological tumour response, disease recurrence, overall survival, and health related quality of life. Exploratory endpoints include translational studies on predictive and prognostic biomarkers.

**Discussion:**

The aim of this study is to select the most promising among three preoperative treatment arms in patients with resectable gastric adenocarcinoma. This treatment regimen will subsequently be compared with the standard therapy in a phase III trial.

**Trial registration:**

clinicaltrials.gov NCT02931890; registered 13 October 2016. Date of first enrolment: 21 December 2017.

## Background

Gastric cancer is the fifth most common malignancy worldwide [1]. In Western countries, gastric cancer patients often present with advanced disease. The 5-year survival rate after surgery alone in resectable gastric cancer is 20–35% [2, 3]. To improve these poor outcomes, different strategies have been evaluated.

Based on the Dutch D1D2 trial, D2 lymphadenectomy with removal of at least 15 lymph nodes is the current recommended surgical approach in patients with potentially curable gastric cancer [4].

In addition to a more extended resection, several (neo-)adjuvant treatments have been evaluated in phase II and III trials. An overview of all published and ongoing randomised (neo-)adjuvant clinical trials in resectable gastric and/or gastro-oesophageal junction (GOJ) cancer published since 1990 is provided in Table 1.

**Table 1:** Overview of all published and ongoing randomised (neo-)adjuvant phase II/phase III clinical trials in patients with resectable gastric cancer and/or GOJ cancer (published from 1990), in order of key-publication

Author	Year key- publication	Year of accrual	Study code/ acronym	Phase (n=)	Design	Treatment	Eligibility	Results	Location
https://clinicaltrials.gov/ct2/show/NCT02931890		2017-present	CRITICS-II	Phase II (*n* = 207)	CT→S CT + CRT→S CRT→S	4×DOC→D2 2×DOC→45Gy + 5×PCa→D2 45Gy + 5×PCa→D2	Adenocarcinoma of the stomach, stage IB-IIIC	In progress	The Netherlands
https://clinicaltrials.gov/ct2/show/NCT02661971		2016-present	FLOT7/RAMSES	Phase-II/III (*n* = 908)	CT→S→CT CT + IT→S→CT + IT	4×FLOT→S→4×FLOT 4×FLOT + R→S→4×FLOT + R	Adenocarcinoma of the stomach or GOJ, cT2 any N, any T N+	In progress	Germany
https://clinicaltrials.gov/ct2/show/NCT01761461		2013-present	ARTIST-II	Phase-II (900)	S→CT S→CT S→CT→CRT→CT	D2→8×S-1 D2→8×S-1 + O D2→2×S-1 + O→45Gy + S-1→4×S-1 + O	Adenocarcinoma of the stomach or GOJ, stage II-III N+	In progress	Korea
https://clinicaltrials.gov/ct2/show/NCT01924819		2009-present	TOPGEAR	Phase III	CT→S→CT CT→CRT→S→CT	3×ECF/ECX→D2→3×ECF/ECX 2×ECF→45Gy + 5FU/X→D2→3×ECF	Adenocarcinoma of the stomach or GOJ, stage IB-IIIC	In progress	Australia Europe
https://clinicaltrials.gov/ct2/show/NCT00216034		2005-2016	HKIT-GC	Phase III (*n* = 280)	S→CT S→CT + IT	S→15×S-1 S→15×S-1 + PSK (12 months)	Adenocarcinoma of the stomach, stage II, IIIA	In progress	Japan
Cats/Jansen et al. [11]	2018	2007–2015	CRITICS	Phase III (*n* = 788)	CT→S→CT CT→S→CRT	3×EOX/ECX→D1+→3×EOX/ECX 3×EOX/ECX→D1+→45Gy + CX	Adenocarcinoma of the stomach or GOJ, stage II-IV (M0)	No difference in OS	The Netherlands Scandinavia
Fuchs et al. [29]	2017	2002–2009	CALGB 80101 (Alliance)	Phase III (*n* = 546)	S→CT→CRT→CT S→CT→CRT→CT	S→5×5FU + LV→45Gy + 5FU→10×5FU + LV S→1×ECF→45Gy + 5FU→2×ECF	Adenocarcinoma of the stomach or GOJ, stage IB-IV (M0)	No difference in OS	USA
Moon et al. [30]	2017	1997–2003		(*n* = 229)	S S→CT S→CT S→CT	S S→5FU (12 months) S→5’DFUR (12 months) S→UFT(12 months)	Adenocarcinoma of the stomach, stage IB-IIIA	No difference in OS	Japan
Cunningham et al. [31]	2017	2007–2014	UK Medical Research Council ST03	Phase II/III (*n* = 1063)	CT→S→CT CT + IT→S→CT + IT	3×ECX→S→3×ECX 3×ECX + B→S→3×ECX + B	Adenocarcinoma of the stomach	No difference in OS	UK
Yoshikawa et al. [32]	2016	2009–2011	COMPASS	Phase II (*n* = 83)	CT→S CT→S CT→S CT→S	2×SC + S 4×SC + S 2×PC + S 4×PC + S	Adenocarcinoma of the stomach, stage III	No difference in OS	Japan
Hashemzadeh et al. [33]	2014	2011–2014		Phase III (*n* = 60)	S CT→S	S 6×DCF→S	Adenocarcinoma of the stomach, stage II-IIIB	Improvement of operability (OS not mentioned)	Iran
Tsuburaya et al. [34]	2014	2004–2009	SAMIT	Phase III (*n* = 1495)	S→CT S→CT S→CT→CT S→CT→CT	D2→12×UFT D2→16×S-1 D2→8×P→9×UFT D2→8×P→12×S-1	Adenocarcinoma of the stomach, stage T4a or T4b	S-1 better OS than UFT. No improvement of OS with sequential therapy	Japan
Kang et al. [35]	2013	2001–2007	AMC0101	Phase III (*n* = 640)	S→CT S→ipCT→CT→CT	D2→2×MMC→5’DFUR(3 months) D2→1x ipC→1×MMC→6×C + 5’DFUR (12 months)	Adenocarcinoma of the stomach, stage I-IV (M0)	Improvement of OS by iaCT	Korea
Tatebe et al. [36]	2013	2005–2009		Phase II (*n* = 73)	S→CT S→CT	D2→8×S-1 (daily during 28 days, 14 days rest) D2→16×S-1 (alternate days during 15 months) (both total 224 days)	Carcinoma of the stomach, stage II-IIIB	No difference in OS, increased treatment compliance in arm 2	Japan
Kang et al. [37]	2013	2002–2006	AMC0201	Phase III (*n* = 855)	S→CT S→CT	D2→2×MMC→5’DFUR(3 months) D2→1×MMC→6×C→5’DFUR(12 months)	Adenocarcinoma of the stomach, stage II-IV (M0)	No difference in OS	Japan
Kim et al. [38]	2012	2002–2006		Phase III	S→CT S→CRT	D2→5×(5FU + LV) D2→1×(5FU + LV)→45Gy + 5FU + LV→2×(5FU + LV)	Adenocarcinoma of the stomach	No difference in OS	Korea
Lee et al. [39]	2012	2004–2008	ARTIST	Phase III (*n* = 458)	S→CT S→CT→CRT	D2→6×XC D2→2×XC→45Gy + X→2×XC	Adenocarcinoma of the stomach, stage IB-IV (M0)	No difference in OS	Korea
Bang et al. [40]	2012	2006–2009	CLASSIC	Phase III (*n* = 1035)	S S→CT	D2 D2→8×CAPOX	Adenocarcinoma of the stomach, stage II-IIIB	Improvement of OS by postoperative CT	Korea China Taiwan
Schumacher et al. [41]	2010	1999–2004	EORTC 40,954	Phase III (*n* = 144) Closed due to insufficient accrual	S CT→S	D2 2×(C + 6×5FU)→D2	Adenocarcinoma of the stomach or GOJ, stage II or III	No difference in OS, higher R0 rate	Germany
Kulig et al. [42]	2010	1995–1999		Phase III (*n* = 309)	S S→CT	S S→3×EAC	Adenocarcinoma of the stomach, M0	No difference in OS	Poland
Bamias et al. [43]	2010	2002–2005		Phase III (*n* = 147)	S→CT S→CRT	S→6×DC/Ca S→45Gy + 6×DC/Ca	Adenocarcinoma of the stomach, M0	No difference in OS	Greece
Biffi et al. [44]	2010	1999–2005	SAKK32/99	Phase III (*n* = 69) Closed due to insufficient accrual	CT→S S→CT	4×DCF→S S→4×DCF	Adenocarcinoma of the stomach, stage IB-IV (M0)	No difference in OS	Italy Switzerland UK France
Schwartz et al. [45]	2009	2001–2004	RTOG-0114	Phase II (*n* = 78)	S→CRT S→CRT	S→2× PCF→45Gy + 5FU + P S→2× PC→45Gy + C + P	Adenocarcinoma of the stomach, stage IB-IIIB	DFS higher in arm 2, arm 2 has too high toxicity rates	USA
Di Constanzo et al. [46]	2008	1995–2000		Phase III (*n* = 258)	S S→CT	S S→4×PELF	Adenocarcinoma of the stomach, stage IB-IV (M0)	No difference in OS	Italia
Jeung et al. [47]	2007	1984–1989		Phase III (*n* = 292)	S→CT S→CT + IT	D2/3→12×DOC + 5FU (18 months) D2/3→12×DOC + 12xpolyA:U + 5FU (18 months)	Adenocarcinoma of the stomach, curatively resected	Improved OS in the CT + IT group	Korea
Nakijima et al. [48]	2007	1997–2001		Phase III (*n* = 190)	S S→CT	D2 D2→UFT (16 months)	Adenocarcinoma of the stomach, curatively resected	Improved OS in the CT group	Japan
De Vita et al. [49]	2007	1996–2001	GOIM 9602 Study	Phase III (*n* = 228)	S S→CT	S S→6×ELFE	Adenocarcinoma of the stomach or GOJ, stage IB-IIIB	No difference in OS	Italy
Cascinu et al. [50]	2007	1998–2003		Phase III (*n* = 201)	S S→CT	S→6×(5FU + LV) S→8×PELFw	High risk adenocarcinoma of the stomach, stage pT3 N0/pT2/pT3N+	No difference in OS	Italy
Sakuramoto et al. [51]	2007	2001–2004	ACTS-GC	Phase III (*n* = 1059)	S S→CT	D2 D2→S-1(12months)	Carcinoma of the stomach, stage II-IIIB	Improvement of OS in the CT group	Japan
Nishikawa et al. [52]	2006 (key publication 2001 in Japanese)	1987–1990	JRFMTC Study no. 10	Phase III (*n* = 1410)	S→CT S→CT	S→1×MMC + UFT (three capsules; 6 months) S→5×MMC + UFT (six capsules; 6 months)	Stomach carcinoma with (sub)serosal invasion	No difference in OS	Japan
Bouché et al. [53]	2005	1989–1997	8801	Phase III (*n* = 260) Closed due to insufficient accrual	S S→CT	D2 D2→1×5FU→4×(5FU + C)	Adenocarcinoma of the stomach; R0; positive lymph nodes and/or T3/T4 tumour	No difference in OS	France
Nashimoto et al. [54]	2003	1993–1994	JOCG 9206–1	Phase III (*n* = 252)	S S→CT	D2 D2→6×(MMC + 5FU + AraC)→oral 5FU (18 months)	Adenocarcinoma of the stomach; N2 or less, macroscopically serosa negative	No difference in OS	Japan
Macdonald et al. [2]	2001	1991–1998	SWOG-Intergroup 0116	Phase III (*n* = 559)	S S→CRT	S S→45Gy + 5FU + LV	Adenocarcinoma of the stomach or GOJ, stage IB-IV(M0)	Improvement of OS in the CRT group	USA
Cirera et al. [55]	1999	1988–1994		Phase III (*n* = 148)	S S→CT	S S→1×MMC→oral 5FU (3 months)	Adenocarcinoma of the stomach, stage III	Improved OS in CT group	Spain
Nakajima et al. [56]	1999	1988–1992		Phase III (*n* = 579)	S S→CT	S S→6×(MMC + 5FU)→ oral UFT (18 months)	Adenocarcinoma of the stomach, stage T1N+ or T2	No difference in OS	Japan
Lise et al. [57]	1995	1980–1989		Phase III (*n* = 314)	S S→CT	S S→7×(5-FU + DOX + MMC)	Adenocarcinoma of the stomach, stage II or III	No difference in OS	Belgium France Germany Italy The Netherlands Portugal Spain Switzerland
Macdonald et al. [58]	1995	1978–1991	A Southwest Oncology Group Study	Phase III (*n* = 193)	S S→CT	S S→6×FAM	Gastric carcinoma, stage I-III	No difference in OS	USA

*S* surgery, *D*2 surgery+D2 lymph node dissection, *CT* chemotherapy, *CRT* chemoradiotherapy,  *ipCT *intraperitoneal CT *IT* immune therapy

*5’DFUR* doxifluridine, *5-FU *5-fluorouracil, *Ara-C* cytarabine, *B* bevacizumab, *C* cisplatin, *CAPOX *capecitabine+oxaliplatin, *CX* cisplatin+capecitabine, *DC/Ca *docetaxel+cisplatin/carboplatin, *DCF* docetaxel+cisplatin+5FU, *DOC* docetaxel+oxaliplatin+capecitabine, *DOX* doxorubicin, *EAC* etoposide+doxorubicin+cisplatin, *ECF* epirubicin+cisplatin+5FU,* ECX *epirubicin+cisplatin+capecitabine, *ELFE* epirubicin+leucovorin+5FU+etoposide, *EOX* epirubicin+oxaliplatin+capecitabine, *FAM* 5-FU+doxorubicin+mitomycin-C, *FLOT* 5FU+folinic acid+oxaliplatin+docetaxel, *ipC* intraperitoneal cisplatin, *LV* leucovorin, *MMC *mitomycin C,* O *oxaliplatin, *P* paclitaxel, *PC *paclitaxel+cisplatin, *PCa* paclitaxel+carboplatin, *PCF* paclitaxel+cisplatin+5FU, *PELF* cisplatin+epirubicin+5-FU+leucovorin, *PELFw* 5FU+epidoxorubicin+leucovorin+cisplatin, *polyA:U *polyadenylicpolyuridylic acid, *PSK *Krestin, *R* ramucirumab, *S-1 *combination tegafur/gimeracil/oteracil, *SC* S-1+cisplatin, *UFT* uracil/tegafur, *X* capecitabine, *XC *capecitabine+cisplatin

In the SWOG/Intergroup trial, 556 patients with resectable gastric or GOJ cancer were randomised between surgery alone versus surgery plus postoperative chemoradiotherapy (CRT) (45 Gy plus 5-fluorouracil (5-FU) and leucovorin). The CRT arm showed significantly improved overall survival (OS) [5]. The three-year OS rates were 41% in the surgery alone group compared to 50% in the CRT group [5]. An updated analysis showed persistent benefit from adjuvant CRT [6].

In 2005, the final results of the MAGIC trial were presented. In this trial, 503 patients with resectable adenocarcinoma of the stomach, GOJ or lower oesophagus were randomised to either perioperative chemotherapy (CT) or surgery alone. Chemotherapy consisted of 3 preoperative and 3 postoperative cycles of epirubicin, cisplatin and 5-FU (ECF). This perioperative regimen of ECF significantly decreased tumour size and induced downstaging. The five-year OS improved significantly from 23% in the surgery alone group to 36% in the perioperative CT group [7].

Many studies have investigated the effect of postoperative CT on survival rates. A meta-analysis from the GASTRIC Group, published in 2010, revealed a significant survival benefit favouring postoperative fluorouracil regimens with a hazard ratio of 0.82 (95% confidence interval (95%CI) 0.76–0.90; *p* < 0.001). The five-year OS increased from 49.6 to 55.3% with postoperative CT. It should be noted that a substantial number of the 17 included studies were carried out in Asia, where patient populations, tumour characteristics and surgical procedures are different compared to the Western world [8].

Current European guidelines include multiple (neo-)adjuvant treatments for patients with resectable gastric cancer [9]. The CRITICS-study was designed to compare OS between patients treated with preoperative CT followed by surgery and postoperative CT versus postoperative CRT [10]. Postoperative CRT did not improve OS as compared to postoperative CT after adequate preoperative CT and surgery [11]. Hence, multiple treatment options remain currently available for patients with locally advanced, resectable gastric cancer. Which patients benefit from which (neo)adjuvant strategy should be addressed in future clinical trials.

There are several important issues that should be addressed in such future studies; which form the rationale behind the CRITICS-II trial. First of all, in most (neo)adjuvant studies patient compliance is low, especially in the postoperative phase (Table 2): 40–60% of patients are not able to complete treatment, mostly due to toxicity, disease progression or patient refusal. Second, there is need for more effective (neo-) adjuvant treatment with equal/less toxicity compared to the widely used epirubicin containing CT. Replacing epirubicin by docetaxel seems to be more effective [12–14], and is considered safe and tolerable [15]. Third, preoperative treatment increases the likelihood of tumour downsizing/downstaging and to achieve surgical radical (R0) resection, as shown in the MAGIC and the CROSS trials [7, 16]. An overview article reported 70–100% radical (R0) resections and a pathological Complete Reponse (pCR) of 7–29% in patients with resectable gastric cancer, preoperatively treated with CRT [17].

**Table 2:** Patient compliance in various recent or ongoing clinical trials in resectable gastric cancer

Study [reference]	Treatment arm	Completed (%)
SWOG [5]	S→CRT	64%
MAGIC [7]	CT→S→CT	42%
ACTS-GC [51]	S→CT	66%
CLASSIC [40]	S→CT	67%
ARTIST [59]	S→CT	75%
	S→CRT	82%
ST03 [31]	CT→S→CT	40%
	CT + B→S→CT + B	37%
TOPGEAR part 1 [27]	CT→S→CT	58%
	CT→CRT→S→CT	45%
FLOT4-AIO [14]	CT→S→CT (3×ECF/ECX)	37%
	CT→S→CT (4×FLOT)	50%
CRITICS [11]	CT→S→CT	46%
	CT→S→CRT	51%

*CT* chemotherapy, *CRT* chemoradiotherapy, *S* surgery, *B* bevacizumab

Finally, preoperative (C)RT allows a more accurate definition of the radiation target volume and margins compared to postoperative (C)RT, which may potentially limit toxicity. For oesophageal cancer, preoperative CRT showed improved survival with acceptable toxicity rates and > 90% patients being able to complete the entire treatment [16].

Based on these considerations, the aim of the current study is to optimise preoperative treatment by comparing three neo-adjuvant treatment modalities: (1) CT, (2) CT plus CRT, and (3) CRT.

## Methods

### Study design and objectives

**Fig. 1:**
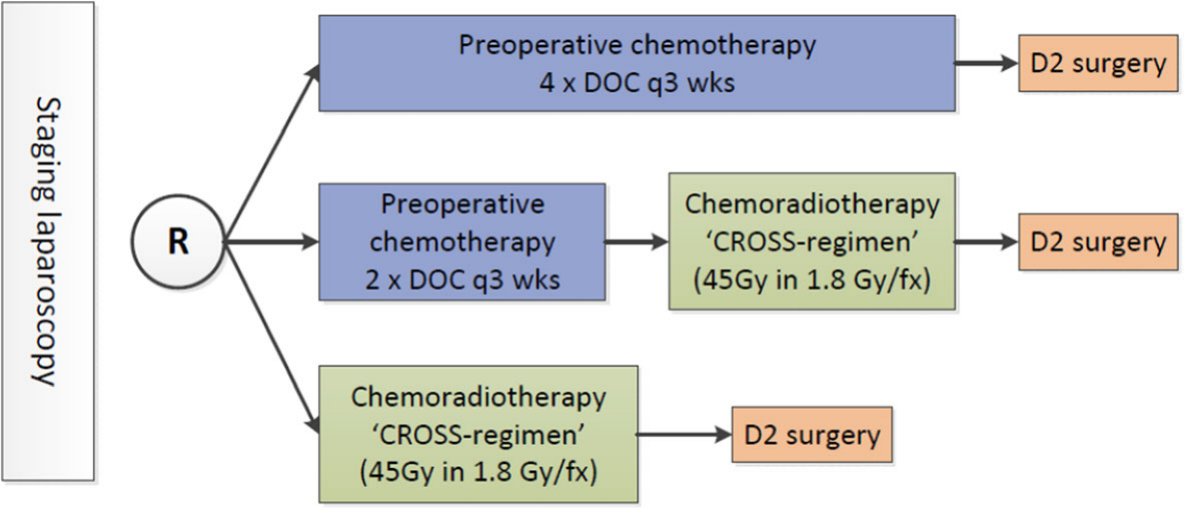
Randomisation scheme CRITICS-II trial

The CRITICS-II study is a multicentre, non-comparative randomised phase II trial. The study is currently recruiting in several centres in The Netherlands. Randomisation is computer generated and will be performed and registered by the data managers. Stratification factors include Lauren classification (intestinal, diffuse, unclassifiable) and centre. The primary objective is to assess 1 year event-free survival in patients treated with preoperative CT, preoperative CT followed by CRT, or preoperative CRT (Fig. 1).

Event-free survival (EFS) is defined as interval between randomisation and local and/or regional recurrence or progression, distant recurrence or death from any cause.

Secondary endpoints are: toxicity, percentage radical (R0) resections, pathological tumour response, disease recurrence, overall survival and health related quality of life (HRQOL). Exploratory endpoints include translational studies into the relationship between classical histological and clinical parameters, the identification of new biomarkers that predict clinical outcome and response, genomic changes in circulating tumour-derived DNA for diagnosis of different molecular subtypes of gastric cancer, and as biomarkers for response to treatment.

### Patient selection and preoperative staging

Patients will be informed and treated by their treating physician. Patients with histologically proven, stage IB-IIIC (TNM 8th edition), resectable gastric adenocarcinoma are eligible for this study. Patients with tumours at the gastro-oesophageal junction (GOJ) may be included, but the tumour bulk has to be in the stomach. Patients should be ≥18 years old and should have WHO performance status < 2. Patients must have adequate haematological, renal and liver function. A staging laparoscopy is mandatory for all patients. At staging laparoscopy, biopsies from suspicious peritoneal lesions and/or free peritoneal fluid if any, should be pathologically proven tumour negative. Patients should have caloric intake ≥1500 kcal/day, verified by a dietician before registration. If caloric intake is < 1500 kcal/day or if bodyweight has decreased > 10% over the last 6 months or > 5% over the last month, dietary intervention such as oral nutritional support or via an enteral feeding tube is mandatory.

Exclusion criteria include: T1 N0 disease (assessed by endoscopic ultrasound), distant metastasis, inoperable/irresectable patients, previous malignancy, solitary functioning kidney within the potential radiation field and gastro-oesophageal stent within the radiation field. Required baseline investigations prior to randomisation consist of blood tests, dietician visit, oesophagoduodenoscopy with representative tumour biopsy samples, computed tomography of the chest and abdomen, staging laparoscopy, renography if there are signs on computed tomography abdomen and/or biochemically signs of impaired renal function. Endoscopic ultrasound (for >T1 N0 disease) and performing a FDG-PET/ computed tomography scan are optional per centre.

### Treatment arms

All treatment arms will start within 15 working days after randomisation. Patients in arm 1 receive four cycles of docetaxel+oxaliplatin+capecitabine (DOC) at a three-weekly interval preoperatively. Patients in arm 2 receive two cycles of DOC at a three-weekly interval, followed by CRT (weekly paclitaxel and carboplatin concurrent with radiotherapy). Chemoradiotherapy starts 3 weeks after start of the second DOC cycle. Patients in arm 3 receive CRT. Prior to surgery, a computed tomography will be performed to exclude progressive disease.

Patients can withdraw their informed consent at any time for any reason if they wish so without any consequences. The investigator can decide to withdraw a patient from the study for urgent medical reasons.

### Preoperative study treatment: Chemotherapy

Chemotherapeutic treatment consists of docetaxel 50 mg/m^2^ on day 1, followed by oxaliplatin 100 mg/m^2^ on day one, followed by capecitabine 850 mg/m^2^ b.i.d. orally on days 1–14. All drugs are administered in a cycle of 21 days. For capecitabine, drug tablet return and a diary are provided. Dose could be discontinued/reduced in case of severe toxicities.

### Preoperative study treatment: Chemoradiotherapy

Radiotherapy consists of 45 Gy in 25 fractions of 1.8 Gy, 5 fractions per week for 5 weeks using image guided intensity modulated radiotherapy/volumic arc therapy (IMRT/VMAT) techniques. The *Clinical Target Volume* (CTV) has to be delineated on CT-images based on all available diagnostic information and should include the tumour, stomach and first draining lymph node stations. For quality assurance, a treatment delineation atlas is available and planning audits are performed. All delineations will be centrally reviewed and if necessary corrected before start of treatment.

Concurrent with radiotherapy, weekly CT is administered. Paclitaxel at a dose of 50 mg/m^2^ and carboplatin Area Under the Curve (AUC) = 2 are given by intravenous infusion on days 1, 8, 15, 22 and 29. Radiotherapy starts at the first day of the first cycle of CT. Radiotherapy and/or chemotherapy dose could be discontinued/reduced in case of severe toxicities.

### Surgery

Surgery is planned 3–4 weeks after preoperative treatment in arm 1 and 6–8 weeks after preoperative treatment in arms 2 and 3. The standard surgical procedure is a (sub)total gastrectomy with a D2 lymph node dissection. A minimum of 15 lymph nodes should be removed. Lymph nodes will be submitted in separate pots, or alternatively, will be clearly marked at the resection specimen. Surgical technique is either open or laparoscopic.

### Pathology

The pathology report includes at least the following items: tumour type, localisation, size of tumour, surgical margins, response to neo-adjuvant therapy in the primary tumour and the lymph nodes (by nodal station), presence of lymphatic invasion, presence of venous invasion, surgical stage, number of (positive) lymph nodes.

All tumours are classified using the Lauren classification (intestinal, diffuse and unclassifiable). For staging, the TNM 8th edition is used. Biopsies, photographs, resection specimen and when performed fresh frozen specimen will be sent for central pathological review. Pathological response according to the Becker scoring system [18] and the Mandard scoring system [19] will be investigated in central pathological review.

### Quality of life

Health related quality of life will be carried out in cooperation with the Prospective Observational Cohort study of Oesophagogastric cancer Patients (POCOP), which is a prospective nationwide study to investigate HRQL in oesophagogastric cancer of all stages [20]. Health related quality of life will be assessed at baseline (before start preoperative treatment), pre-surgery and from surgery every 3 months in the first year, every 6 months in the second year and yearly thereafter until 5 years.

### Translational research: Circulating tumour DNA (ctDNA)

Patients can optionally participate in this part of the protocol. All patients participating in this part of the protocol need to have signed informed consent specifically for this optional side study of the trial. Blood will be collected at baseline before start of any of the preoperative treatment modalities, after every preoperative treatment modality, after surgery and at every follow-up visit until 5 years after surgery or until recurrence. Material processing and storage will be done centrally at the VU University Medical Center.

### Toxicity and (serious) adverse events

Toxicity during this trial is scored using the Common Terminology Criteria for Adverse Events (CTCAE) version 4.0. Serious adverse events are defined according to the rules of good clinical practice and must be reported within one working day and are reported once yearly in the annual safety report. The Clavien-Dindo grading system is used for the classification of surgical complications [21].

### Follow-up

After treatment, patients are followed by all treating medical specialists. The follow-up moments in the first year are, counted from surgery, at 1, 3, 6 and 12 months. From the second year, follow-up will occur every 6 months until 5 years after surgery. To enable evaluation of the primary endpoint, one-year EFS, CT chest and abdomen will be performed 1 year after randomisation.

### Protocol amendments

Future protocol modifications will be submitted as amendment at the central medical ethics committee. After obtaining approval, this will be communicated to the participating sites by the study coordinator or project manager.

### Data management and responsibilities

The central data management, data processing and statistical analysis of this study are performed at the Biometric Department of the sponsor. The study coordinators are responsible, in cooperation with the Data Centre, for writing the protocol, reviewing all case report forms, reporting and correctness of SAE. They are also responsible for answering clinical questions, treatment and evaluation of the patients and for publishing study results. Authors on the key-publication includes at least the protocol writing committee and additional a maximum of three authors per centre. The key-publication will be submitted to a major, peer-reviewed journal. Data is entered by data managers into a coded electronic case-report form. The study will be considered as a medium risk according Dutch Federation of University Medical Centres guidelines. Site monitoring will be performed by an independent Clinical Research Monitor or the person to whom the monitoring tasks have been delegated. The monitor will judge: compliance with the protocol, all applicable regulatory requirements, informed consent, source data verification, investigator study file, SAE/Serious Unexpected Serious Adverse Reactions. If necessary, active feedback will be provided to the participating sites. The study will be monitored and an auditing trail will not be performed routinely. The study coordinators will have access to the final dataset. Queries will be sent in case of missing data.

### Statistics

The primary endpoint of this trial is EFS at 1 year. Statistical analysis will be performed on basis of intention to treat analysis. For each treatment arm, a 1-year EFS of 60% is considered insufficient and a 1-year EFS of 75% is active enough to further explore in a phase III study, taking other endpoints in consideration. The 1-year EFS of 60% is based on results of the MAGIC [22] and the CRITICS trial [11]. The design calculations assume Weibull distributions (shape = 1), which is equivalent to an exponential distribution. Scale parameters were chosen such that 1 year EFS would be 60% and 75% respectively. The type I (α) error is fixed at 10% (one-sided) and power is set at 90%.The R package OptInterim minimising the expected sample size was used for calculating the sample size for each stage as well as the time of interim and final analyses. We used exact binomial correction adjusting all sample size and analysis times.

The trial is designed as a two-stage trial with one interim analysis at which futile arms are discontinued. Because the primary endpoint is evaluated at 1 year, the commonly used Simon’s two stage design would imply up to 1 year in which the trial would be put on hold. To improve the efficiency, we will use the two stage design for survival endpoints as proposed by Case and Morgan [23].

The expected accrual is 30 patients per arm per year. After accruing 42 patients in each arm, the interim analysis will be performed. Futile arms will be discontinued and the remaining arms will accrue 27 more patients each (to a total of 69 patients in each arm). Final analysis will be performed 1 year after accrual of last patient. In both stages the null hypothesis will be evaluated using Lin’s statistics [24]. If in the final analysis more than one arm will reject the null hypothesis, the decision about which arm should continue to the phase III trial will be made based on the Kaplan Meier point estimates of the 1-year EFS in combination with other factors such as toxicity, cost, convenience and quality of life.

## Discussion

Here we describe the study protocol of the CRITICS-II trial, a clinical phase II study aimed at identifying a new and optimal preoperative treatment in patients with resectable gastric cancer by comparing three preoperative treatment arms. Several considerations have led to the design of this trial. First, patients’ compliance, especially in the postoperative phase of the treatment, is low (Table 2). Second, there is need for a more effective (neo)adjuvant treatment [12–14], that is why we incorporated a docetaxel containing CT regimen in this trial. Third, preoperative treatment increases the likelihood of tumour downsizing/downstaging and to achieve tumour free resection margins [7, 16]. The last consideration is that preoperative (chemo)radiotherapy allows a more accurate definition of the radiation target volume and margins, which may potentially limit toxicity in patients receiving preoperative CRT.

Epirubicin containing CT regimens (epirubicin+cisplatin/oxaliplatin+capecitabine; ECX/EOX) are widely used in the preoperative setting, as recommended by European guidelines [9]. The choice to incorporate DOC as CT regimen was based on several studies.

Naj Mohammad et al. investigated efficacy and safety of triplet versus doublet CT in locally advanced or metastatic oesophagogastric carcinoma. When subgroups were examined, especially triplet CT with taxane, cisplatin and fluoropyrimidine revealed significant benefit [13]. Roth et al. published in 2007 the results of a phase II trial among 121 patients. Patients were randomised between docetaxel+cisplatin+5FU (DCF) and ECF. The regimen containing DCF seemed to be more effective than ECF. DCF had shorter time-to-response, which may suggest that DCF is more favourable as preoperative treatment compared to ECF. However, DCF showed a trend towards increased myelosuppression and infectious complications [12]. Van Deenen et al. published in 2015 the results of a phase Ia/Ib trial of DOC in patients with advanced cancer of the stomach/GOJ. The data showed that the combination of intravenous docetaxel 50 mg/m2 and oxaliplatin 100 mg/m2 on day 1 plus capecitabine 850 mg/m2 b.i.d. for 14 days in 3-week cycles were safe, tolerable and effective [15]. Toxicities among 28 patients were frequent, as is often the case in triple CT regimen, but remained non-severe and well manageable in most patients. The most common grade ≥ 3 toxicities were leukocytopenia (15%) and neutropenia (24%). Febrile neutropenia occurred in 12% of the patients, which is less than the 29% reported for the combination of DCF, reported by van Cutsem et al. [25].

Recently, the results of the FLOT4-AIO have been presented at the American Society of Clinical Oncology annual meeting [14]. In this study, patients with resectable gastric cancer or GOJ cancer, were randomised between perioperative CT with ECF/ECX versus fluorouracil+leucovorin+oxaliplatin+docetaxel (FLOT). Perioperative CT with ECF/ECX contained three pre- and postoperative cycles and perioperative CT with FLOT contained four pre- and postoperative cycles. Survival rates were significantly improved in the FLOT arm. This study showed significantly improved survival rates in the FLOT arm. Once the key-publication is available, it is expected that European guidelines will be updated. Various Dutch centres have already implemented the FLOT-regimen as standard perioperative CT. The CT regimen used in the CRITCS-II trial is a slight variation of FLOT, and the results of the FLOT4-AIO study support the use of DOC as preoperative CT in our study.

So far, this is the third randomised controlled trial in which patients with upper GI cancer are treated with preoperative CRT. The CROSS study forms the basis for the chosen CRT schedule [16]. One of the main concerns of preoperative CRT is the potential increased risk for surgical complications such as anastomotic leakage. The CROSS study showed no difference in postoperative complication rate between preoperative CRT and surgery alone [16]. Currently, the TOPGEAR study is recruiting patients. In this study, patients with resectable gastric or GOJ cancer are 1:1 randomised to receive either preoperative CT or preoperative CT followed by CRT, then followed by surgery and postoperative CT [26]. Recently, the interim analysis of the TOPGEAR trial has been published, which included a total of 120 patients. It revealed no difference in surgical complications between both groups [27]. A retrospective analysis from a prospectively maintained database from the *MD Anderson Cancer Center* was published in 2017. This analysis included a total of 346 patients with resectable gastric cancer, of whom 44% underwent preoperative CRT [28]. No significant association between type of preoperative therapy and the risk of anastomotic leakage was found.

### Future perspectives

The aim of this study is to select the most promising preoperative treatment regimen among three experimental arms in patients with resectable gastric adenocarcinoma. This treatment regimen will subsequently be compared with the current standard therapy in a phase III trial.

## Abbreviations

5-FU: 5-FluoroUracil
AUC: Area Under the Curve
AVL: Antoni van Leeuwenhoek
b.i.d.: Bis in die (twice daily)
CRITICS: ChemoRadiotherapy after Induction chemoTherapy In Cancer of the Stomach
CROSS: ChemoRadiotherapy for Oesophageal cancer followed by Surgery Study
CRT: ChemoRadioTherapy
CT: ChemoTherapy
CTCAE: Common Toxicity Criteria for Adverse Events
CTV: Clinical Target Volume
D1: Dissection 1: lymph node stations 1–7
D2: Dissection 2: lymph node stations 8–12
DCF: Docetaxel Cisplatin 5-FU
DFS: Disease-Free Survival
DOC: Docetaxel Oxaliplatin Capecitabine
ECF: Epirubicin Cisplatin 5-Flurouracil
EFS: Event-Free Survival
FLOT: Fluorouracil/Leucovorin/Oxaliplatin/Docetaxel
GASTRIC: Global Advanced/Adjuvant Stomach Tumor Research International Collaboration
GOJ: Gastro-Oesophageal Junction
Gy: Gray
HRQOL: Health Related Quality of Life
IMRT: Intensity Modulated RadioTherapy
MAGIC: Medical Research Council Adjuvant Gastric Infusional Chemotherapy
METC: Medical Ethics Committee
OS: Overall Survival
POCOP: Prospective Observational Cohort Study of Oesophageal-gastric cancer Patients
R0: Free Resection Margin
SWOG: South West Oncology Group
TNM: Tumour Nodes Metastasis
TOPGEAR: Trial Of Preoperative therapy for Gastric and Esophagiagstric junction AdenocaRcinoma
VMAT: VoluMetric Arc Therapy
WHO: World Health Organisation

## References

[1] Ferlay J, Soerjomataram I, Dikshit R, Eser S, Mathers C, Rebelo M (2015). Cancer incidence and mortality worldwide: sources, methods and major patterns in GLOBOCAN 2012. Int J Cancer.

[2] De Angelis R, Sant M, Coleman MP, Francisci S, Baili P, Pierannunzio D, et al. Cancer survival in Europe 1999-2007 by country and age: results of EUROCARE--5-a population-based study. Lancet Oncol. 2014;15(1):23–34. 10.1016/S1470-2045(13)70546-1 24314615

[3] Msika S, Benhamiche AM, Jouve J-L, Rat P, Faivre J (2000). Prognostic factors after curative resection for gastric cancer. A population-based study. Eur J Cancer.

[4] Songun I, Putter H, Kranenbarg EMK, Sasako M, van de Velde CJH (2010). Surgical treatment of gastric cancer: 15-year follow-up results of the randomised nationwide Dutch D1D2 trial. Lancet Oncol.

[5] Macdonald JS, Smalley SR, Benedetti J, Hundahl SA, Estes NC, Stemmermann GN (2001). Chemoradiotherapy after surgery compared with surgery alone for adenocarcinoma of the stomach or gastroesophageal junction. N Engl J Med.

[6] Smalley SR, Benedetti JK, Haller DG, Hundahl SA, Estes NC, Ajani JA (2012). Updated analysis of SWOG-directed intergroup study 0116: a phase III trial of adjuvant radiochemotherapy versus observation after curative gastric cancer resection. J Clin Oncol.

[7] Cunningham D, Allum WH, Stenning SP, Thompson JN, Van de Velde CJ, Nicolson M (2006). Perioperative chemotherapy versus surgery alone for Resectable Gastroesophageal Cancer. N Engl J Med.

[8] Paoletti X, Oba K, Burzykowski T, Michiels S, Ohashi Y, Pignon JP (2010). Benefit of adjuvant chemotherapy for Resectable gastric Cancer. J Am Med Assoc.

[9] Smyth EC, Verheij M, Allum W, Cunningham D, Cervantes A, Arnold D (2016). Gastric cancer: ESMO clinical practice guidelines for diagnosis, treatment and follow-up. Ann Oncol.

[10] Dikken JL, van Sandick JW, Maurits Swellengrebel H, Lind PA, Putter H, Jansen EP (2011). Neo-adjuvant chemotherapy followed by surgery and chemotherapy or by surgery and chemoradiotherapy for patients with resectable gastric cancer (CRITICS). BMC Cancer.

[11] Cats A, Jansen EPM, van Grieken NCT, Sikorska K, Lind P, Nordsmark M (2018). Chemotherapy versus chemoradiotherapy after surgery and preoperative chemotherapy for resectable gastric cancer (CRITICS): an international, open-label, randomised phase 3 trial. Lancet Oncol.

[12] Roth AD, Fazio N, Stupp R, Falk S, Bernhard J, Saletti P (2007). Docetaxel, cisplatin, and fluorouracil; docetaxel and cisplatin; and epirubicin, cisplatin, and fluorouracil as systemic treatment for advanced gastric carcinoma: a randomized phase II trial of the Swiss group for clinical cancer research. J Clin Oncol.

[13] Haj Mohammad N, ter Veer E, Ngai L, Mali R, van Oijen MGH, van Laarhoven HWM (2015). Optimal first-line chemotherapeutic treatment in patients with locally advanced or metastatic esophagogastric carcinoma: triplet versus doublet chemotherapy: a systematic literature review and meta-analysis. Cancer Metastasis Rev.

[14] Al-Batran SE, Hopmann N, Schmalenberg H, Kopp H-G, Haag GM, Luley KB (2017). Perioperative chemotherapy with docetaxel, oxaliplatin, and fluorouracil/leucovorin (FLOT) versus epirubicin, cisplatin, and fluorouracil or capecitabine (ECF/ECX) for resectable gastric or gastroesophageal junction (GEJ) adenocarcinoma (FLOT4-AIO). A mul. J Clin Oncol.

[15] Deenen MJ, Meulendijks D, Boot H, Legdeur MCJC, Beijnen JH, Schellens JHM (2015). Phase 1a/1b and pharmacogenetic study of docetaxel, oxaliplatin and capecitabine in patients with advanced cancer of the stomach or the gastroesophageal junction. Cancer Chemother Pharmacol.

[16] van Hagen P, Hulshof MCCM, van Lanschot JJB, Steyerberg EW, van B Henegouwen MI, Wijnhoven BPL (2012). Preoperative Chemoradiotherapy for esophageal or Junctional Cancer. N Engl J Med.

[17] Trip AK, Verheij M, van Sandick JW, Boot H, Jansen EP M, Cats A (2015). Emerging issues in multimodality treatment of gastric cancer. Transl Gastrointest Cancer.

[18] Becker K, Mueller JD, Schulmacher C, Ott K, Fink U, Busch R (2003). Histomorphology and grading of regression in gastric carcinoma treated with neoadjuvant chemotherapy. Cancer.

[19] Mandard A-M, Dalibard F, Mandard J-C, Marnay J, Henry-Amar M, Petiot J-F (1994). Pathologic assessment of tumor regression after preoperative chemoradiotherapy of esophageal carcinoma. Clinicopathologic correlations. Cancer.

[20] van den Braak RRJ C, van Rijssen LB, van Kleef JJ, Vink GR, Berbee M, van Berge Henegouwen MI (2018). Nationwide comprehensive gastro-intestinal cancer cohorts: the 3P initiative. Acta Oncol.

[21] Dindo D, Demartines N, Clavien PA (2004). Classification of surgical complications: a new proposal with evaluation in a cohort of 6336 patients and results of a survey. Ann Surg.

[22] Cunningham D, Starling N, Rao S, Iveson T, Nicolson M, Coxon F (2008). Capecitabine and Oxaliplatin for advanced Esophagogastric Cancer. N Engl J Med.

[23] Case LD, Morgan TM (2003). Design of Phase II cancer trials evaluating survival probabilities. BMC Med Res Methodol.

[24] Lin DY, Shen L, Ying Z, Breslow NE (1996). Group sequential designs for monitoring survival probabilities. Biometrics.

[25] Van Cutsem E, Moiseyenko VM, Tjulandin S, Majlis A, Constenla M, Boni C (2006). Phase III study of Docetaxel and Cisplatin plus fluorouracil compared with Cisplatin and fluorouracil as first-line therapy for advanced gastric Cancer: a report of the V325 study group. J Clin Oncol.

[26] Leong T, Smithers BM, Michael M, Gebski V, Boussioutas A, Miller D (2015). TOPGEAR: a randomised phase III trial of perioperative ECF chemotherapy versus preoperative chemoradiation plus perioperative ECF chemotherapy for resectable gastric cancer (an international, intergroup trial of the AGITG/TROG/EORTC/NCIC CTG). BMC Cancer.

[27] Leong T, Smithers BM, Haustermans K, Michael M, Gebski V, Miller D (2017). TOPGEAR: a randomized, phase III trial of perioperative ECF chemotherapy with or without preoperative Chemoradiation for Resectable gastric Cancer: interim results from an international, intergroup trial of the AGITG, TROG, EORTC and CCTG. Ann Surg Oncol.

[28] Ikoma N, Das P, Blum M, Estrella JS, Devine CE, Wang X (2017). Preoperative Chemoradiation therapy does not increase risk of anastomotic leak in patients with gastric Cancer. Int J Radiat Oncol Biol Phys.

[29] Fuchs CS, Enzinger PC, Meyerhardt J, Mayer RJ, Mamon HJ, Swanson RS (2017). Adjuvant chemoradiotlherapy with epirubicin, cisplatin, and fluorouracil compared with adjuvant chemoradiotherapy with fluorouracil and leucovorin after curative resection of gastric cancer: Results from CALGB 80101 (alliance). J Clin Oncol.

[30] Moon JH, Fijiwara Y, Hirao M, Imamura H, Kimura Y, Fijitani K (2017). Randomized controlled trial of adjuvant chemotherapy with Fluoropyrimidines versus surgery-alone for gastric Cancer. Anticancer Res.

[31] Cunningham D, Stenning SP, Smyth EC, Okines AF, Allum WH, Rowley S (2017). Peri-operative chemotherapy with or without bevacizumab in operable oesophagogastric adenocarcinoma (UK medical research council ST03): primary analysis results of a multicentre, open-label, randomised phase 2–3 trial. Lancet Oncol..

[32] Yoshikawa T, Morita S, Tanabe K, Nishikawa K, Ito Y, Matsui T (2016). Survival results of a randomised two-by-two factorial phase II trial comparing neoadjuvant chemotherapy with two and four courses of S-1 plus cisplatin (SC) and paclitaxel plus cisplatin (PC) followed by D2 gastrectomy for resectable advanced gastric canc. Eur J Cancer.

[33] Hashemzadeh S, Pourzand A, Somi MH, Zarrintan S, Javad-Rashid R, Esfahani A (2014). The effects of neoadjuvant chemotherapy on resectability of locally-advanced gastric adenocarcinoma: a clinical trial. Int J Surg.

[34] Tsuburaya A, Yoshida K, Kobayashi M, Yoshino S, Takahashi M, Takiguchi N (2014). Sequential paclitaxel followed by tegafur and uracil (UFT) or S-1 versus UFT or S-1 monotherapy as adjuvant chemotherapy for T4a/b gastric cancer (SAMIT): a phase 3 factorial randomised controlled trial. Lancet Oncol..

[35] Kang Y-K, Yook JH, Chang H-M, Ryu M-H, Yoo C, Zang DY (2014). Enhanced efficacy of postoperative adjuvant chemotherapy in advanced gastric cancer: results from a phase 3 randomized trial (AMC0101). Cancer Chemother Pharmacol.

[36] Tatebe S, Tsujitani S, Nakamura S, Shimizu T, Yamane N, Nishidoi H (2014). Feasibility study of alternate-day S-1 as adjuvant chemotherapy for gastric cancer: a randomized controlled trial. Gastric Cancer.

[37] Kang YK, Chang HM, Yook JH, Ryu MH, Park I, Min YJ (2013). Adjuvant chemotherapy for gastric cancer: a randomised phase 3 trial of mitomycin-C plus either short-term doxifluridine or long-term doxifluridine plus cisplatin after curative D2 gastrectomy (AMC0201). Br J Cancer.

[38] Kim TH, Park SR, Ryu KW, Kim YW, Bae JM, Lee JH (2012). Phase 3 trial of postoperative chemotherapy alone versus chemoradiation therapy in stage III-IV gastric cancer treated with R0 gastrectomy and D2 lymph node dissection. Int J Radiat Oncol Biol Phys.

[39] Lee J, Lim DH, Kim S, Park SH, Park JO, Park YS (2012). Phase III trial comparing capecitabine plus cisplatin versus capecitabine plus cisplatin with concurrent capecitabine radiotherapy in completely resected gastric cancer with D2 lymph node dissection: the ARTIST trial. J Clin Oncol.

[40] Bang YJ, Kim YW, Yang HK, Chung HC, Park YK, Lee KH (2012). Adjuvant capecitabine and oxaliplatin for gastric cancer after D2 gastrectomy (CLASSIC): a phase 3 open-label, randomised controlled trial. Lancet.

[41] Schuhmacher C, Gretschel S, Lordick F, Reichardt P, Hohenberger W, Eisenberger CF (2010). Neoadjuvant chemotherapy compared with surgery alone for locally advanced cancer of the stomach and cardia: European organisation for research and treatment of cancer randomized trial 40954. J Clin Oncol.

[42] Kulig J, Kolodziejczyk P, Sierzega M, Bobrzynski L, Jedrys J, Popiela T (2010). Adjuvant chemotherapy with etoposide, adriamycin and cisplatin compared with surgery alone in the treatment of gastric cancer: a phase III randomized, multicenter, clinical trial. Oncology.

[43] Bamias A, Karina M, Papakostas P, Kostopoulos I, Bobos M, Vourli G (2010). A randomized phase iii study of adjuvant platinum/docetaxel chemotherapy with or without radiation therapy in patients with gastric cancer. Cancer Chemother Pharmacol.

[44] Biffi R, Fazio N, Luca F, Chiappa A, Andreoni B, Zampino MG (2010). Surgical outcome after docetaxel-based neoadjuvant chemotherapy in locally-advanced gastric cancer. World J Gastroenterol.

[45] Schwartz GK, Winter K, Minsky BD, Crane C, Thomson PJ, Anne P (2009). Randomized phase II trial evaluating two paclitaxel and cisplatin-containing chemoradiation regimens as adjuvant therapy in resected gastric cancer (RTOG-0114). J Clin Oncol.

[46] Di Costanzo F, Gasperoni S, Manzione L, Bisagni G, Labianca R, Bravi S (2008). Adjuvant chemotherapy in completely resected gastric cancer: a randomized phase III trial conducted by GOIRC. J Natl Cancer Inst.

[47] Jeung HC, Moon YW, Rha SY, Yoo NC, Roh JK, Noh SH (2008). Phase III trial of adjuvant 5-fluorouracil and adriamycin versus 5-fluorouracil, adriamycin, and polyadenylic-polyuridylic acid (poly a:U) for locally advanced gastric cancer after curative surgery: final results of 15-year follow-up. Ann Oncol.

[48] Nakajima T, Kinoshita T, Nashimoto A, Sairenji M, Yamaguchi T, Sakamoto J (2007). Randomized controlled trial of adjuvant uracil-tegafur versus surgery alone for serosa-negative, locally advanced gastric cancer. Br J Surg.

[49] De Vita F, Giuliani F, Orditura M, Maiello E, Galizia G, Di Martino N (2007). Adjuvant chemotherapy with epirubicin, leucovorin, 5-fluorouracil and etoposide regimen in resected gastric cancer patients: a randomized phase III trial by the Gruppo Oncologico Italia Meridionale (GOIM 9602 study). Ann Oncol.

[50] Cascinu S, Labianca R, Barone C, Santoro A, Carnaghi C, Cassano A (2007). Adjuvant treatment of high-risk, radically resected gastric cancer patients with 5-fluorouracil, leucovorin, cisplatin, and epidoxorubicin in a randomized controlled trial. J Natl Cancer Inst.

[51] Sakuramoto S, Sasako M, Yamaguchi T, Kinoshita T, Fujii M, Nashimoto A (2007). Adjuvant chemotherapy for gastric Cancer with S-1, an oral Fluoropyrimidine. N Engl J Med.

[52] Nishikawa T, Maetani S (2007). Evaluation of intensive adjuvant chemotherapy in gastric cancer using life expectancy compared with log-rank test as a measure of survival benefit. Ann Surg Oncol.

[53] Bouché O, Ychou M, Burtin P, Bedenne L, Ducreux M, Lebreton G (2005). Adjuvant chemotherapy with 5-fluorouracil and cisplatin compared with surgery alone for gastric cancer: 7-year results of the FFCD randomized phase III trial (8801). Ann Oncol.

[54] Nashimoto A, Nakajima T, Furukawa H, Kitamura M, Kinoshita T, Yamamura Y (2003). Randomized trial of adjuvant chemotherapy with mitomycin, fluorouracil, and cytosine arabinoside followed by oral fluorouracil in serosa-negative gastric cancer: Japan clinical oncology group 9206-1. J Clin Oncol.

[55] Cirera L, Balil A, Batiste-Alentorn E, Tusquets I, Cardona T, Urcusa A (1999). Randomized clinical trial of adjuvant Mytomycin plus Tegafur in patients with resected stage III gastric Cancer. J Clin Oncol.

[56] Nakajima T, Nashimoto A, Kitamura M, Kito T, Iwanaga T, Okabayashi K (1999). Adjuvant mitomycin and fluorouracil followed by oral uracil plus tegafur in serosa-negative gastric cancer: a randomised trial. Lancet.

[57] Lise M, Nitti D, Marchet A, Sahmoud T, Buyse M, Duez N (1995). Final results of a phase III clinical trial of adjuvant chemotherapy with the modified fluorouracil, doxorubicin, and mitomycin regimen in resectable gastric cancer. J Clin Oncol.

[58] Macdonald JS, Fleming TR, Peterson RF, Berenberg JL, McClure S, Chapman RA (1995). Adjuvant chemotherapy with 5-FU, adriamycin, mitomycin-C (FAM) versus surgery alone for patients with locally advanced gastric adenocarcinoma: a southwest oncology group study. Ann Surg Oncol.

[59] Park SH, Sohn TS, Lee J, Lim DH, Hong ME, Kim KM (2015). Phase III trial to compare adjuvant chemotherapy with capecitabine and cisplatin versus concurrent chemoradiotherapy in gastric cancer: final report of the adjuvant chemoradiotherapy in stomach tumors trial, including survival and subset analyses. J Clin Oncol.

